# Significant decline in anticancer immune capacity during puberty in the Tasmanian devil

**DOI:** 10.1038/srep44716

**Published:** 2017-03-16

**Authors:** Yuanyuan Cheng, Kim Heasman, Sarah Peck, Emma Peel, Rebecca M. Gooley, Anthony T. Papenfuss, Carolyn J. Hogg, Katherine Belov

**Affiliations:** 1 School of Life and Environmental Sciences, The University of Sydney, New South Wales, 2006, Australia; 2 Department of Primary Industries, Parks, Water and Environment, 134 Macquarie Street, Hobart, Tasmania, 7000, Australia; 3 Bioinformatics Division, The Walter and Eliza Hall Institute of Medical Research, Parkville, Victoria, 3052, Australia; 4 Computational Cancer Biology Program, Peter MacCallum Cancer Centre, Victoria, 3000, Australia; 5 Department of Medical Biology, University of Melbourne, Victoria, 3010, Australia; 6 Sir Peter MacCallum Department of Oncology, University of Melbourne, Victoria, 3010, Australia

## Abstract

Tasmanian devils (*Sarcophilus harrisii*) are at risk of extinction in the wild due to Devil Facial Tumour Disease (DFTD), a rare contagious cancer. The prevalence of DFTD differs by age class: higher disease prevalence is seen in adults (2–3 years) versus younger devils (<2 years). Here we propose that immunological changes during puberty may play a role in susceptibility to DFTD. We show that the second year of life is a key developmental period for Tasmanian devils, during which they undergo puberty and pronounced changes in the immune system. Puberty coincides with a significant decrease in lymphocyte abundance resulting in a much higher neutrophil:lymphocyte ratio in adults than subadults. Quantitative PCR analysis of gene expression of transcription factors T-bet and GATA-3 and cytokines interferon gamma (IFN-γ) and interleukin 4 (IL-4) revealed a drastic increase in GATA-3 and IL-4 expression during puberty. These changes led to a significantly lower IFN-γ:IL-4 ratio in 2-year-olds than <1 year olds (on average 1.3-fold difference in males and 4.0-fold in females), which reflects a major shift of the immune system towards Th2 responses. These results all indicate that adult devils are expected to have a lower anticancer immune capacity than subadults, which may explain the observed pattern of disease prevalence of DFTD in the wild.

The Tasmanian devil (*Sarcophilus harrisii*), the largest living carnivorous marsupial in the world, is on the brink of extinction due to a contagious cancer known as Devil Facial Tumour Disease (DFTD)^1^. The tumour originally arose from Schwann cells in a female devil, and has subsequently been transmitted between devils as allograft primarily through biting^2^,^3^. By suppressing the expression of Major Histocompatibility Complex (MHC) molecules on the cell surface, the tumour cells evade host immune surveillance and trigger no immune response in most affected animals^4^,^5^. So far, DFTD has spread across the majority of devil population, causing up to 97% local population declines^6^.

DFTD has been found to have a significant impact on the demographic structure and life history of devils^1^,^7^. Prior to DFTD the devil’s life expectancy was up to six years in the wild, with females starting to breed after two years of age^7^. In DFTD-affected populations, animals aged three or above become rare as older devils succumb to the disease, and females start to give birth at an earlier age^7^. Here we test the hypothesis that the significantly higher disease prevalence in adult devils (2–3 years) than in younger animals (<2 years)^8^ could be driven by changes in the immune system which occur during puberty.

Puberty and associated changes in sex hormone levels are known to influence the development and function of the immune system^9^,^10^,^11^. In humans and other model species, sex hormones have been shown to affect cytokine profiles and responses^12^, whilst puberty is associated with marked changes in white blood cell populations^9^. In the devil, although reproductive physiology and seasonality have been studied^8^,^13^, timing of puberty and its impact on the immune system has not been examined. Here, we investigated changes in devil’s immune system during puberty, with a focus on main leukocyte populations (neutrophils and lymphocytes) and key T helper cell cytokines (interferon gamma and interleukin 4) in the blood.

## Results and Discussion

### Pubertal maturation in the devil

The timing of puberty in devils was investigated by monitoring changes in the circulating level of progesterone or testosterone in eight subadult captive animals (four female, four male) across four time points: 11 months, 14 months, 16 months, and 20 months of age, corresponding to the months of March (early autumn), June (early winter), August (late winter), and December (early summer) in a single year.

**Figure 1:**
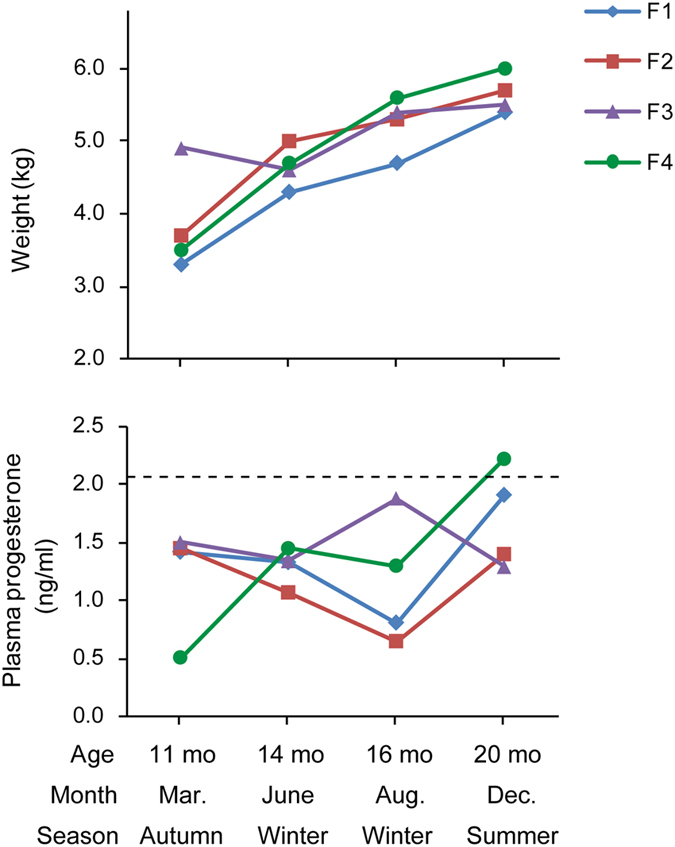
Changes in weight and plasma progesterone concentration in four female subadult devils. The dashed line represents the average progesterone level in adult females (n = 8; 2–4 yr) in December at the same captive facility.

In subadult females, consistent trends in the plasma progesterone concentration over the examined period were observed between two individuals (Fig. 1; F1 and F2), though the other two devils showed different fluctuation patterns. In most of the animals studied, the plasma concentration of progesterone was higher at 11 months (March) of age than 14 months (June), coinciding with the breeding season which peaks around March and ends by June^14^. Later in the year, the level increased substantially between August (16 months of age) and December (20 months), leading towards the breeding season of the following year. These observations suggest that although female devils usually have their first litter in the third year of life (i.e. at age 2)^7^, their progesterone level has already started to synchronise with the breeding season when they are one year old. It is also noteworthy that in December, all four examined 20-month-old devils had a progesterone level falling within the range seen in adult females (1.08–3.04 ng/ml), which possibly indicates that female devils have reached sexual maturity and can become reproductively active by late age one. This is supported by previous observations of precocious breeding by 1-year-old female devils in DFTD-affected populations^7^ and in a re-introduced population (Maria Island; CJH, personal communication). It was also noticed that the progesterone concentrations of devils observed in this study were higher than those previously reported for a different captive facility^13^. This may suggest that, as is seen in humans^15^, mean levels of reproductive hormones are variable between devil populations, which could be caused by a wide range of nutritional, demographic, or ecological factors.

Subadult male devils aged less than two showed low plasma testosterone levels at all four examined time points (Table 1). The concentration was mostly below the detectable threshold (0.04 ng/ml) of the assay used, except for during the peak period of the mating season in March, when a small surge occurred in most individuals. This might be due to episodic fluctuations of the testosterone level during puberty, with peaks falling outside most of the sampled time points. Due to high variability of testosterone concentrations in devils throughout the year (0.02–0.74 ng/ml)^8^, it is difficult to infer when puberty occurs in male devils solely based on testosterone levels. But given previous evidence demonstrating that 2-year-old males have fully developed reproductive system and active spermatogenesis^8^, and our empirical data suggesting a 60% average breeding success rate in captive 2-year-old males, male devils are likely to reach full sexual maturity at age two.

### Increase in neutrophil:lymphocyte ratio

**Figure 2:**
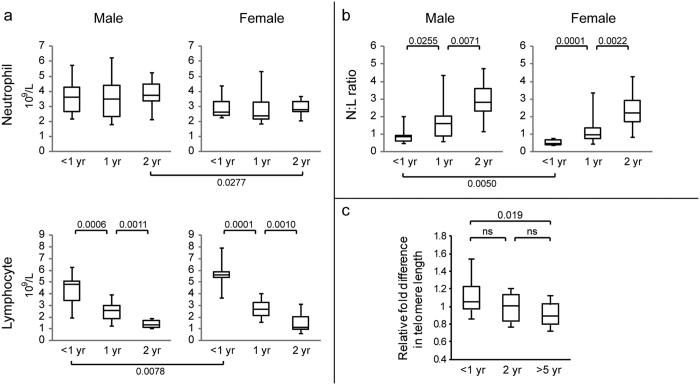
Age-related changes in (**a**) neutrophil and lymphocyte counts, (**b**) neutrophil:lymphocyte ratio, and (**c**) telomere length in the peripheral blood in devils. Mann-Whitney U tests were performed for pairs of sample groups; p-values smaller than 0.05 are shown.

Neutrophils and lymphocytes comprise the majority (94.0% ± 3.8%) of the leukocyte population in the peripheral blood of devils. Absolute neutrophil counts remained relatively stable among three examined age groups (Fig. 2): <1 year old (10 males, 8 females), 1-year-olds (18 males, 15 females), and 2-year-olds (7 males, 10 females). Consistent with previous observations^16^,^17^, male devils had higher neutrophil counts than females, though the difference was not significant until age two, whereas female devils under one year old had higher lymphocyte counts than males. The abundance of lymphocytes appeared to decline significantly during puberty in both male and female devils. Similar observations have been made in human adolescent boys^9^. This lymphocyte decline in devils was not the result of loss of cell division capacity due to telomere shortening as is seen in progressive aging^18^, as there was no significant reduction of the peripheral blood telomere length between <1 and 2-year age groups (Fig. 2). Instead, based on our recent observation using computed tomography indicating that the thymus of young devils starts to regress before 10 months of age (KB, personal communication), lowered naïve T cell production by the thymus has likely contributed to the observed decline in lymphocyte abundance.

Shrinkage of the lymphocyte population led to a substantial increase in the neutrophil:lymphocyte (N:L) ratio in devils through puberty (Fig. 2). The peripheral blood N:L ratio, which reflects the balance between the innate and adaptive immunity, is known to be an indicator of antitumour efficacy of the host immune system^19^,^20^. Many tumours are characterised by the influx of T cell suppressive myeloid cells such as neutrophils, which can hinder T cell mediated anticancer responses^21^. High N:L ratios are a significant predictor of lower rates of patient survival in many human cancers^22^,^23^. The ratio has been shown to positively correlate with age in humans, rising from 1.53 ± 0.56 in the younger group (<20 years) to 1.99 ± 0.60 in the >70 year old group^24^. In the devils examined, the N:L ratio increased from 0.92 ± 0.44 to 2.94 ± 1.26 in males, and from 0.53 ± 0.15 to 2.40 ± 1.15 in females, between ages of less than one year and two years. Taken together haematology data reported previously^17^,^25^, N:L ratios greater than two are common in adult devils. Such high baseline N:L ratios may have played a role in causing the high susceptibility of the species to neoplasms^26^,^27^. Moreover, the significant increase in the N:L ratio through puberty provides another possible explanation for the elevated DFTD susceptibility in adult devils compared to subadults^1^.

### Decrease in IFN-γ:IL-4 ratio

**Figure 3:**
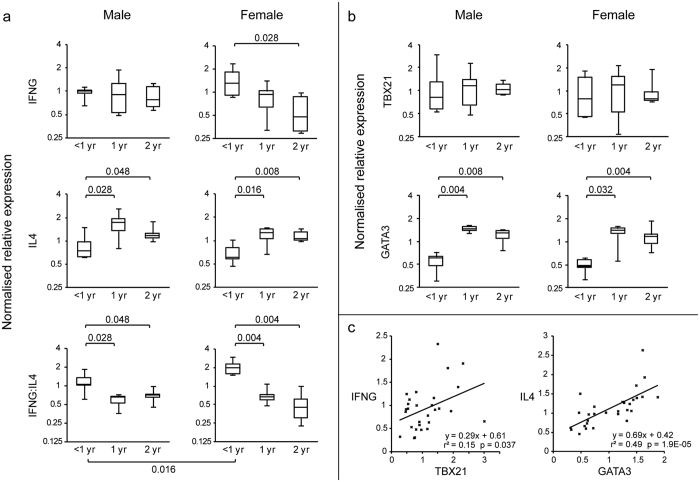
Age-related changes in Th1/Th2 gene expression in the peripheral blood in devils. (**a**) Relative quantification of the expression of cytokines IFN-γ and IL-4; (**b**) Relative quantification of the expression of transcription factors T-bet and GATA-3; (**c**) Association between T-bet and IFN-γ gene expression, and between GATA-3 and IL-4 expression. Mann-Whitney U tests were performed for pairs of sample groups; p-values smaller than 0.05 are shown (panel a and b).

Cytokines interferon gamma (IFN-γ) and interleukin 4 (IL-4) are centrally involved in the regulation of cellular and humoral immune responses. In female devils, the expression level of IFN-γ in the blood showed a drastic decline during puberty (Fig. 3a), whereas in males, IFN-γ was produced at similar levels among the three examined age groups (five males and five females studied per age group). By contrast, the production of IL-4 changed significantly in both male and female devils: the expression level was the lowest in young devils (<1 year), and then rose rapidly during age one to reach a relatively higher level in adult devils. Similar patterns were observed in the gene expression of GATA-3 (Fig. 3b), a transcription factor that directly regulates the expression of IL-4^28^, with a tight positive correlation (r^2^ = 0.49, p = 1.9 × 10^−5^) detected between GATA-3 and IL-4 levels (Fig. 3c). Transcription factor T-bet (TBX21), which controls IFN-γ production^29^, showed no age-related change in gene expression, though a significant association (r^2^ = 0.15, p = 0.037) was found between T-bet and IFN-γ expression levels across all samples.

Sex hormones are known to have immunomodulatory effects and can influence lymphocyte differentiation and cytokine production, though the exact effect can be context and concentration-dependent and sometimes variable between species. For example, testosterone has been found to stimulate CD4^+^ T cell secretion of IL-10 in mice^30^, but shows an inhibitory effect on IL-10 production in humans^31^. Testosterone can also suppress the expression of IFN-γ^31^, whereas dehydroepiandrosterone, another androgen crucial for male characteristics development, appears to have the opposite effect^32^. Estradiol at preovulatory concentrations increases IFN-γ level; however, at elevated concentrations (e.g. during pregnancy or with estradiol treatment), it reduces IFN-γ and induces IL-4 production significantly^33^,^34^. Similarly, progesterone has been shown to inhibit the expression of T-bet in pregnant cows, though this effect was not observed in non-pregnant cows^35^. Therefore, drastic changes in sex hormone levels during puberty likely have played a role in altering the cytokine profile in subadult devils, though further investigation will be needed to dissect the mechanisms involved.

Changes in IFN-γ and IL-4 levels resulted in significantly lower IFN-γ:IL-4 ratios in 1-year-old and 2-year-old devils than younger animals (Fig. 3a). While IFN-γ is a hallmark cytokine of T helper 1 (Th1) responses, which promotes cellular immunity against cancerous or infected (by viruses or intracellular pathogens) cells, IL-4 drives naïve T cells to differentiate towards T helper 2 (Th2) cells, skewing the immune system towards humoral responses to extracellular pathogens and allergens^36^,^37^,^38^. In many cancers, eradiation of tumour cells largely relies on cytotoxic T cells and natural killer cells activated via Th1 pathways, which can be impeded by IL-4 and other Th2 cytokines^39^,^40^. IL-4 can also augment tumour cell production of IL-10 (Th2)^41^, further inhibiting recruitment and activity of antitumour cells. In light of this, the sharp decline of IFN-γ:IL-4 ratio at age one in the devil, which reflects a major shift of the immune system towards Th2, is another important sign of anticancer immunity declining during puberty.

## Conclusion

The second year of life is a key developmental period for Tasmanian devils, during which they undergo puberty accompanied by pronounced changes in the immune system. We show that a significant decrease in lymphocyte abundance and alterations in cytokine IFN-γ and IL-4 profiles during puberty lead to a higher neutrophil:lymphocyte ratio and a lower IFN-γ:IL-4 ratio in 2-year-olds than <1 year old young devils. These results explain why adult devils are expected to have lower anticancer immune responses than subadults, which provides a reason for the differences in DFTD disease prevalence in adults and younger animals.

## Materials and Methods

### Sample collection

Blood samples were collected with approval from The University of Sydney Animal Ethics Committee under project number 550, with all experiments performed in accordance with relevant guidelines and regulations. Approximately 1 ml blood per kg body mass (no more than 5 ml in total) was sampled from each animal. Blood was collected and processed differently depending on downstream analyses, which are detailed below.

### Hormone concentration

Serial samples were collected from four female and four male young devils at 11 months, 14 months, 16 months, and 20 months of age for studying changes in the plasma progesterone or testosterone level. Eight female devils aged 2–4 years were also sampled for assessing progesterone concentrations in adults. Blood was collected in BD Vacutainer heparin tubes and plasma was recovered on the same day and stored at -20 °C until radioimmunoassay (RIA). Both testosterone and progesterone assays were carried out using MP Biomedical ImmuChem RIA kits as per protocol in duplicate and counted in a LKB CliniGamma counter. Results were calculated using AssayZap. Sensitivity of the progesterone assay was 0.075 ng/ml with an Intra-assay coefficient of variation (CV) of High Control (9.5 ng/ml) 5% and Low Control (1.0 ng/ml) 10.8% and an Inter-assay CV of 8.5% and 11.2% respectively. Sensitivity of the testosterone assay was 0.04 ng/ml with an Intra-assay CV of High Control (4.0 ng/ml) 4.6% and Low Control (0.3 ng/ml) 6% and Inter-assay CV of 7.5% and 12.7% respectively.

### Blood cell count

Blood cell count data was collected from captive devils belonging to three age groups: <1 year old (10 males, 8 females), one year old (18 males, 15 females), and two years old (7 males, 10 females). Previously described protocol^16^ for haematological analysis in the devil was adopted with minor modifications. Briefly, blood was collected in EDTA tubes and were either counted on the same day of collection or preserved with Streck Cell Preservative™, which qualitatively and quantitatively stabilizes leukocyte subsets in the blood, and examined within a week. Lymphocyte and neutrophil counts were performed on a Sysmex XT-2000iV Haematology Analyzer or Sysmex KX21N Haematology Analyzer. Five blood smears were also produced and stained using Rapid Diff (Australian Biostain); these smears were manually counted and showed results consistent with the automated counter. To examine age- and sex-related differences, statistical tests (Mann-Whitney U tests) were performed for pairs of sample groups, that is, between different ages of the same sex, and between males and females of the same age.

### Relative fold difference in telomere length

Relative quantification of peripheral blood telomere length was carried out using previously described telomere specific primers and protocol^42^. Genomic DNA was extracted from EDTA blood of 10 under one year old, 10 two year old, and 12 five year old devils using DNeasy Blood & Tissue Kit (Qiagen). All samples had an A_260_/A_280_ ratio between 1.786 and 2.000. Single-copy reference gene *RPLP0* was used for normalisation^42^. Real-time PCRs were carried out on a RotorGene 6000 in a total volume of 15 μl, containing 7.5 μl 2x Quantifast Sybr Green PCR Master Mix (Qiagen), 0.5 μM each of forward and reverse primers, and approximately 1 ng of gDNA. Samples were analyzed in triplicates with no-template negative controls included in each run. PCR programs comprised an initial step of 95 °C 5 minutes, followed by 40 cycles of two-step cycling of 95 °C for 15 seconds and 60 °C for 30 seconds. A composite standard containing equal parts of gDNA from all samples was made and included in each run. Standard curves were generated using five serial 1:4 dilutions of the standard. Telomere PCR had an efficiency of 1.01 and a correlation coefficient of standard curve (R^2^) 0.990, while *PRLP0* showed efficiency 0.95 and R^2^ 0.997. Normalised relative fold differences in telomere copy number were calculated using the Pfaffl method^43^.

### Relative quantification of IFN-γ and IL-4 gene expression

Relative expression quantification of interferon gamma (*IFNG*) and interleukin 4 (*IL4*) was carried out for 30 devils belonging to three age groups (<1 year, one-year, and two-year), with each group containing five males and five females (different to the animals used for haematology analysis). RNA was extracted from blood preserved in RNAprotect Animal Blood Tubes 500 μl (Qiagen) using RNeasy Protect Animal Blood Kit (Qiagen) with on-column DNase treatment. All samples had an RNA Intergrity Number higher than 9.0 as assessed on a 2100 Bioanalyzer (Agilent Technologies). cDNA was synthesized from 500 ng RNA using SuperScript VILO Master Mix (Invitrogen). Real-time PCR primers for *IFNG, IL4, TBX21,* and *GATA3* were designed using software Oligo v6.71, with forward and reverse primers located on different exons: IFNG-F AGTTCTTCTGGCTGTCTTTCTC, IFNG-R CCCTCTTTCCAAGTCTTCATCA; IL4-F GTCCACGGACAGAGAAGAAACTG, IL4-R ATGTCTAGCACTTCCATCTCAGAG; TBX21-F AGCCTCCAATAATGTGACTCAG, TBX21-R GTGAAGGTGTGGGTCAAAGAG; GATA3-F CACAGGGTTCGGATGTAAGTC, GATA3-R CAGTACCATCTCTCCTCCACAA. Primers for reference genes *GAPDH*^3^ and *GUSB*^44^ were adopted from previous publications. PCRs were carried out on a RotorGene 6000 in a total volume of 20 μl, containing 10 μl 2x Quantifast Sybr Green PCR Master Mix (Qiagen), 0.5 μM each of forward and reverse primers, and approximately 60 ng cDNA. All samples were analyzed in triplicates with no-template negative controls included for each gene in each run. PCR conditions were as follows: an initial step of 95 °C 5 minutes; 40 cycles of two-step cycling of 95 °C for 10 seconds and 60 °C for 30 seconds; a final heating step from 50 °C to 99 °C with fluorescence signal collected every 1 °C to generate a melting curve. A composite standard containing equal parts of cDNA from all samples was made and included in each run. Standard curves were generated for all genes using four to five 1:3 serial dilutions of the standard. PCR efficiencies ranged between 0.97 and 1.02, and R^2^ between 0.986 and 0.999. Normalised relative expression of target genes were calculated using the geNorm equations^45^.

## Additional Information

**How to cite this article**: Cheng, Y. *et al*. Significant decline in anticancer immune capacity during puberty in the Tasmanian devil. *Sci. Rep.* **7**, 44716; doi: 10.1038/srep44716 (2017).

**Publisher's note:** Springer Nature remains neutral with regard to jurisdictional claims in published maps and institutional affiliations.

## Figures and Tables

**Table 1:** Weight and plasma testosterone concentration in four male subadult devils.

Animal ID	Weight (kg)				Testosterone (ng/ml)			
	11 mo (Mar.)	14 mo (June)	16 mo (Aug.)	20 mo (Dec.)	11 mo (Mar.)	14 mo (June)	16 mo (Aug.)	20 mo (Dec.)
M1	3.6	4.9	6	7.3	0.07	<0.04	<0.04	<0.04
M2	4.6	5.9	6.1	7.8	0.08	<0.04	<0.04	<0.04
M3	5.2	6.5	7	8.3	<0.04	<0.04	<0.04	<0.04
M4	3.1	3.7	4.5	5.4	0.04	<0.04	<0.04	<0.04
